# Methylome analyses of three glioblastoma cohorts reveal chemotherapy sensitivity markers within DDR genes

**DOI:** 10.1002/cam4.3447

**Published:** 2020-09-29

**Authors:** Tobias Kessler, Anne Berberich, Ahmed Sadik, Felix Sahm, Thierry Gorlia, Christoph Meisner, Dirk C. Hoffmann, Antje Wick, Philipp Kickingereder, Petra Rübmann, Martin Bendszus, Christiane Opitz, Michael Weller, Martin van den Bent, Roger Stupp, Frank Winkler, Alba Brandes, Andreas von Deimling, Michael Platten, Wolfgang Wick

**Affiliations:** ^1^ Clinical Cooperation Unit Neurooncology German Cancer Consortium (DKTK) German Cancer Research Center (DKFZ) Heidelberg Germany; ^2^ Department of Neurology and Neurooncology Program of the National Center for Tumor Diseases Heidelberg University Hospital Heidelberg Germany; ^3^ Brain Tumor Metabolism DKTK DKFZ Heidelberg Germany; ^4^ Department of Neuropathology Heidelberg University Hospital Heidelberg Germany; ^5^ Clinical Cooperation Unit Neuropathology DKTK DKFZ Heidelberg Germany; ^6^ European Organization for Research and Treatment of Cancer Headquarters Brussels Belgium; ^7^ Institute for Clinical Epidemiology and Biometry Tübingen Germany; ^8^ Faculty of Biosciences Heidelberg University Heidelberg Germany; ^9^ Department of Neuroradiology Heidelberg University Hospital Heidelberg Germany; ^10^ Department of Neurology University Hospital and University of Zurich Zurich Switzerland; ^11^ The Brain Tumor Center Erasmus MC Cancer Institute Rotterdam The Netherlands; ^12^ Feinberg School of Medicine Northwestern University Chicago IL USA; ^13^ Department of Medical Oncology Azienda USL‐IRCCS Institute of Neurological Sciences Bologna Italy; ^14^ Clinical Cooperation Unit Neuroimmunology and Brain Tumor Immunology DKTK DKFZ Heidelberg Germany; ^15^ Department of Neurology Medical Faculty Mannheim MCTN Heidelberg University Mannheim Germany

## Abstract

**Background:**

Gliomas evade current therapies through primary and acquired resistance and the effect of temozolomide is mainly restricted to *methylguanin‐O6-methyltransferase promoter (MGMT)* promoter hypermethylated tumors. Further resistance markers are largely unknown and would help for better stratification.

**Methods:**

Clinical data and methylation profiles from the NOA‐08 (104, elderly glioblastoma) and the EORTC 26101 (297, glioblastoma) studies and 398 patients with glioblastoma from the Heidelberg Neuro‐Oncology center have been analyzed focused on the predictive effect of DNA damage response (DDR) gene methylation. Candidate genes were validated in vitro.

**Results:**

Twenty‐eight glioblastoma 5'‐cytosine‐phosphat‐guanine‐3' (CpGs) from 17 DDR genes negatively correlated with expression and were used together with telomerase reverse transcriptase (TERT) promoter mutations in further analysis. CpG methylation of DDR genes shows highest association with the mesenchymal (MES) and receptor tyrosine kinase (RTK) II glioblastoma subgroup. MES tumors have lower tumor purity compared to RTK I and II subgroup tumors. CpG hypomethylation of *DDR* genes *TP73* and *PRPF19* correlated with worse patient survival in particular in *MGMT* promoter unmethylated tumors. *TERT* promoter mutation is most frequent in RTK I and II subtypes and associated with worse survival. Primary glioma cells show methylation patterns that resemble RTK I and II glioblastoma and long term established glioma cell lines do not match with glioblastoma subtypes. Silencing of selected resistance genes *PRPF19* and *TERT* increase sensitivity to temozolomide in vitro.

**Conclusion:**

Hypomethylation of DDR genes and *TERT* promoter mutations is associated with worse tumor prognosis, dependent on the methylation cluster and *MGMT* promoter methylation status in *IDH* wild‐type glioblastoma.

## Graphical abstract

Hypomethylation of DNA damage response genes correlates with worse survival in glioblastoma patients. Silencing of *PRPF19* and *TERT* in glioblastoma in vitro models increase sensitivity to temozolomide

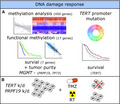

## 1 INTRODUCTION

Diffuse gliomas regularly evade current therapies and several mechanisms behind resistance and sensitivity have not only recently been discovered with high‐throughput efforts demonstrating the molecular faith of gliomas at recurrence,^1^ but also novel targeted approaches proposing a novel radiation sensitivity biomarker.^2^ Classically, methylation of the *methylguanin‐O6-methyltransferase promoter (MGMT)* promoter is known to predict response to alkylating chemotherapy, with at best minimal responses for patients with an unmethylated *MGMT* promoter at diagnosis and progression.^3^, ^4^ The NOA‐08 study demonstrated an impressive survival benefit in *MGMT* promoter methylated elderly patients with temozolomide treatment compared to radiotherapy alone,^5^ but this may potentially be restricted to a specific molecular tissue context.^6^, ^7^

Nevertheless, disease control for more than a few years is achieved in barely 15%‐20% of younger glioblastoma patients with tumors with a hypermethylated *MGMT* promoter only. Therefore, further markers to better stratify patients for treatment response prediction and to decide for chemo‐ or radiotherapy are of great interest. Recent approaches suggested that the DNA damage response (DDR) gene methylome could facilitate to predict resistance vs sensitivity to radio‐ and chemotherapy in World Health Organization grade II, *IDH* mutant gliomas.^8^ These markers have not been validated in an independent data set yet and an effect of differential DDR gene methylation in *IDH* wild‐type glioblastomas dependent on the clinically relevant *MGMT* promoter methylation is unknown. Also, various inhibitors of DDR components are in preclinical and clinical development allowing to further exploit the concept of synthetic lethality.^9^, ^10^

Recent studies suggested a benefit from a methylated *MGMT* promoter when receiving temozolomide chemotherapy only in patients with tumors with additional promoter mutation of the DDR gene telomerase reverse transcriptase (TERT).^11^, ^12^ We identified a subset of glioblastoma patients by methylation clustering, who benefitted most from temozolomide treatment when the *MGMT* promoter is methylated. These patients showed enhanced *TERT* expression.^6^ In addition, *TERT* promoter mutation was found to be one of the earlier but not earliest events of gliomagenesis and leads to increased TERT expression rather than TERT methylation.^13^

Therefore, methylation of DDR genes and mutation status in particular for TERT may alter the sensitivity to standard radiochemotherapy treatment especially in tumors lacking *MGMT* promoter methylation. In these tumors prognostic factors are rarely known and would be important for patient stratification. We here explored the potential of DDR methylation as further prognostic markers. We based or hypothesis on 450 genes that have been identified as DDR genes^14^ and restricted the analysis to genes where methylation negatively impacted expression. Of the DDR genes, TERT is unique in the sense that TERT promoter mutations mainly influence expression, and therefore, for TERT not methylation but the promoter mutations status was included. Based on these assumptions, we aimed to identify prognostic markers of DDR genes in a combined analysis of three large well‐documented glioblastoma cohorts from the NOA‐08^7^ and EORC 26101^15^ studies as well as a patient cohort from our Heidelberg Neuro‐Oncology Center spanning a variety of conditions. Promising candidates are validated in vitro.

## 2 METHODS

### 2.1 Patient cohorts

Two glioma studies and a well‐documented cohort of patients treated in Heidelberg with study grade follow up that was used as an exploratory, hypothesis‐generating data set were included in the present work. Altogether this study involves 799 patients with diffuse *IDH* wild‐type glioma. Regression analyses were performed either for each study separately or combined for all three studies together with correction for the confounding effect of the study, as indicated.

#### 2.1.1 NOA‐08

The NOA‐08 study compared radiotherapy (RT) with temozolomide (TMZ) chemotherapy in elderly patients (age at diagnosis >65 years) with anaplastic astrocytoma or glioblastoma.^5^ The original study population consisted of 373 patients. The investigated biomarker cohort consists of 104 patients (radiotherapy group: 53, temozolomide group: 51).

#### 2.1.2 EORTC 26101

The EORTC 26101 study randomized patients with progressive glioblastoma between bevacizumab (BEV) with lomustine (CCNU) and CCNU alone.^15^ The original study cohort consisted of 596 patients. The investigated biomarker cohort where methylation array data and paraffin tissue for evaluating TERT mutations status was available consisted of 297 patients.

#### 2.1.3 Heidelberg cohort

About 398 patients from the Heidelberg Neuro‐Oncology center diagnosed with *IDH* wild‐type glioblastoma between 07/2014 and 01/2018 based on histopathological and molecular characteristics. This cohort includes 43 patients from the NCT Neuro Master Match (N^2^M^2^) pilot study extensively characterized with whole‐genome sequencing, whole‐exome sequencing (WES), RNAseq, and methylation analysis.^16^

Inclusion of patients into this analysis is covered by a local Heidelberg ethics vote (no. S307/2019).

Patients of the two clinical study biomarker cohorts (NOA‐08 and EORTC‐26101) are comparable to the original study population regarding survival times and treatment (Table 1).

### 2.2 Illumina HumanMethylation450 and HumanMethylationEPIC arrays

The Illumina Infinium HumanMethylation450 (450k) bead chip and MethylationEPIC kits were used to obtain the DNA methylation status at >450 000 and >850 000 5'‐cytosine‐phosphat‐guanine‐3' (CpG) sites, respectively (Illumina), according to the manufacturer's instructions at the Genomics and Proteomics Core Facility of the German Cancer Research Center (DKFZ) in Heidelberg, Germany from fresh frozen and paraffin embedded tissue of study cohorts as well as selected primary patient‐derived cultures. Samples were analyzed using the R (www.r-proje​ct.org) based methylation pipeline “ChAMP” 2.10.1.^17^ In brief, filtering was done for multihit sites, SNPs, and XY chromosome‐related CpGs, then, data were normalized with a BMIQ based method and analyzed for batch effects with a singular value decomposition algorithm. Batch effects related to the tissue used (paraffin embedded [FFPE] vs fresh frozen [KRYO]) were corrected using ComBat. *MGMT* promoter methylation status was determined by the algorithm of Bady et al.^18^ The classifier score and association with specific classifier types (RTK I, II, and MES) was performed using the Neuropathology 2.0 tool described in Capper el al.^19^ Custom scripts based on the R packages “minfi” (version 1.26.2) and “conumee” (version 1.14.0) were implemented for copy‐number variation profiling and visualization.

### 2.3 Tumor purity estimation

Tumor purity estimation was performed on normalized beta values of methylation data with the R package InfiniumPurity version 1.3.1.^20^

### 2.4 Selection of functional DDR gene methylation CpGs

Potential DDR genes were obtained from a 450 putative gene list that was published previously.^14^ CpGs present in the 450k methylation array in the promoter region were tested for negative association with expression in The Cancer Genome Atlas (TCGA, RRID:SCR_003193) data set obtained from the National Cancer Institute Genomic Commons Data Portal (GDC Portal, portal.gdc.cancer.gov). CpGs with a correlation *r* < −.3 and a Benjamini‐Hochberg adjusted *P* < .05 were regarded as “functional” in a sense that high methylation correlates with low RNA expression. About 28 CpGs from 17 genes met this criterion and were used for further analysis. The thereafter used term “functional DDR CpGs” refers to these 28 CpGs.

### 2.5 Cell culture and in vitro assays

A detailed description of in vitro assays is given in the Methods S1. Generation and maintenance of established glioma cell lines and primary glioma cell cultures was performed using standard methods as described previously.^21^, ^22^

### 2.6 Data analysis

Statistical analyses were carried out with R 3.5.1 (www.r-proje​ct.org, RRID:SCR_001905) and Microsoft Excel 2016 (Microsoft Corporation; RRID:SCR_016137). If not otherwise stated, a *P* < .05 was considered as significant and marked with a “*.” No outliers have been excluded. Correction for testing of multiple CpGs or genes was performed using the Benjamini‐Hochberg procedure. The performances of the multivariate proportional hazards models were calculated using a c‐index concordance statistic in R. If not otherwise indicated bar charts show mean values and standard deviation of at least three independent experiments. For survival analysis, we used custom adaptations of the R packages “survival” (version 2.42‐6) and “survminer” (version 0.4.3). A Cox proportional hazards model for univariate and multivariate analysis was used to assess the correlation between CpG methylation and survival as implemented in the “coxph” function. Clustering of methylation array samples was done as described before^6^ using the ConsensusClusterPlus package in R. Dimensionality reduction and network analysis were also performed with R and are described in the Methods S1. All figures were produced using R‐based packages.

### 2.7 Data availability

Methylation raw and processed data are made accessible via the Gene Expression Omnibus (GEO) database (https://www.ncbi.nlm.nih.gov/geo/; RRID:SCR_005012) under the GEO accession numbers GSE12​2920 (NOA‐08 biomarker cohort), GSE14​3755 (EORTC 26101 biomarker cohort), GSE12​2994 and GSE143842 (both Heidelberg cohort). TCGA data from glioblastoma patients can be accessed via CDC portal (portal.gdc.cancer.gov).

**TABLE 1:** Comparison of Biomarker cohorts in the present analysis with the original study cohorts.

	Biomarker cohort	Full study cohort
NOA‐08		
Patient number [n (%)]	104 (28%)	373 (100%)
Overall survival [median (95% CI)]^a^	11.2 (9.6‐13.8)	8.7 (8.0‐9.8)
Event‐free survival [median (95% CI)]^a^	4.1 (3.7‐4.5)	4.4 (3.6‐5.5)
TMZ group [n (%)]	51 (49%)	195 (52%)
RT group [n (%)]	53 (51%)	178 (48%)
EORTC‐26101		
Patient number [n (%)]	297 (50%)	596 (100%)
Lomustine first group [n (%)]	117 (39%)	231 (39%)
BEV ± Lomustine first group [n (%)]	180 (61%)	365 (61%)
Overall survival [median (95% CI)]^a^	8.6 (7.9‐9.9)	8.9 (8.2‐9.6)
Progression‐free survival [median (95% CI)]^a^	3.0 (2.8‐3.7)	2.9 (2.8‐3.0)
Heidelberg cohort		
Patient number [n]	398	NA
Overall survival [median (95% CI)]^a^	24.9 (19.2‐31.0)	NA
Progression‐free survival [median (95% CI)]^a^	8.2 (7.2‐9.2)	NA
Patients with RT + TMZ [n (%)]	252 (63%)	NA

Abbreviations: BEV, bevacizumab; RT, radiotherapy; TMZ, temozolomide.

a Survival times are given in months.

## 3 RESULTS

### 3.1 The functional DDR methylome of IDH wild‐type glioblastoma

**FIGURE 1:**
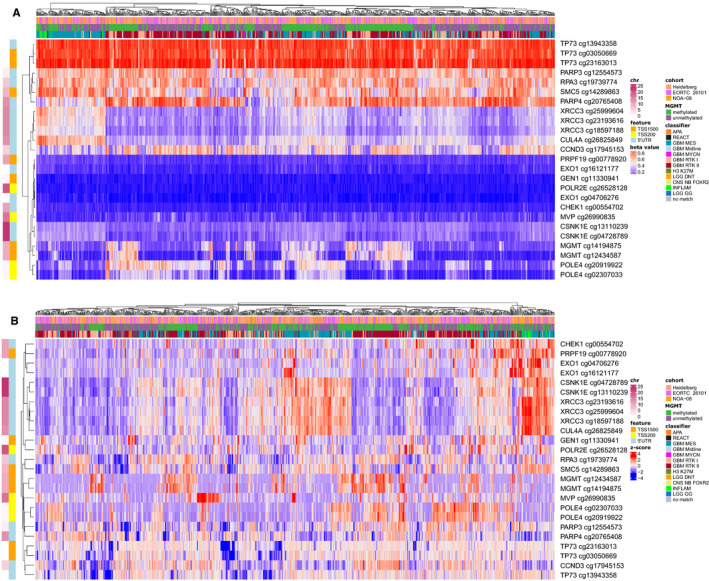
Functional DNA damage response 5'‐cytosine‐phosphat‐guanine‐3' (CpG) methylation in clinical study cohorts. Heatmaps of functional CpGs of all three studies (NOA‐08, n = 104, EORTC 26101 , n = 297 and Heidelberg, n = 398) combined showing normalized methylation beta values (A) and row scaled values (B). 5ʹ‐UTR, 5ʹ untranslated region; chr, chromosome; TSS200, 0‐200 base pairs upstream of transcription start site; TSS1500, 200‐1500 base pairs upstream of transcription start site; a full list of classifier abbreviations can be found in the Supporting Information

Figure S1 shows the workflow of the project and study cohorts used. DDR CpGs were derived from a list of 450 previously published expert‐curated human DDR genes.^14^ We then correlated RNA expression of these genes with CpG methylation in the TCGA glioblastoma data set and negatively correlated CpGs (*r* < −.3, *P*.adj < .05, see Section 2) were regarded as functional and used for further analysis. In total, 28 CpGs from 17 genes were identified (Table S1). Additionally, *TERT* expression correlates with *TERT* promoter mutations in samples from Heidelberg, for which expression and mutation data were available (Figure S2). Mutation frequencies of DDR genes excluding *TERT* were rather low in the TCGA glioblastoma data set with only three genes exceeding a predefined 3% mutation frequency in the TCGA glioblastoma data set (*STAG* [5%], *TP53* [53%], *ATRX* [9%]) all without harboring functional CpGs in their promoter. Similar results were obtained in the WES Heidelberg glioblastoma *IDH* wild‐type cohort (n = 43). Figure 1A,B show a heatmap with methylation values of functional DDR CpGs together with methylation classifier assignments of all 799 *IDH* wild‐type tumors from the EORTC26101, NOA‐08, and Heidelberg cohorts. Hierarchical clustering divides the CpGs into four different groups, those with generally low methylation, high methylation, and two groups with a broad range of methylation values (Figure S3).

**FIGURE 2:**
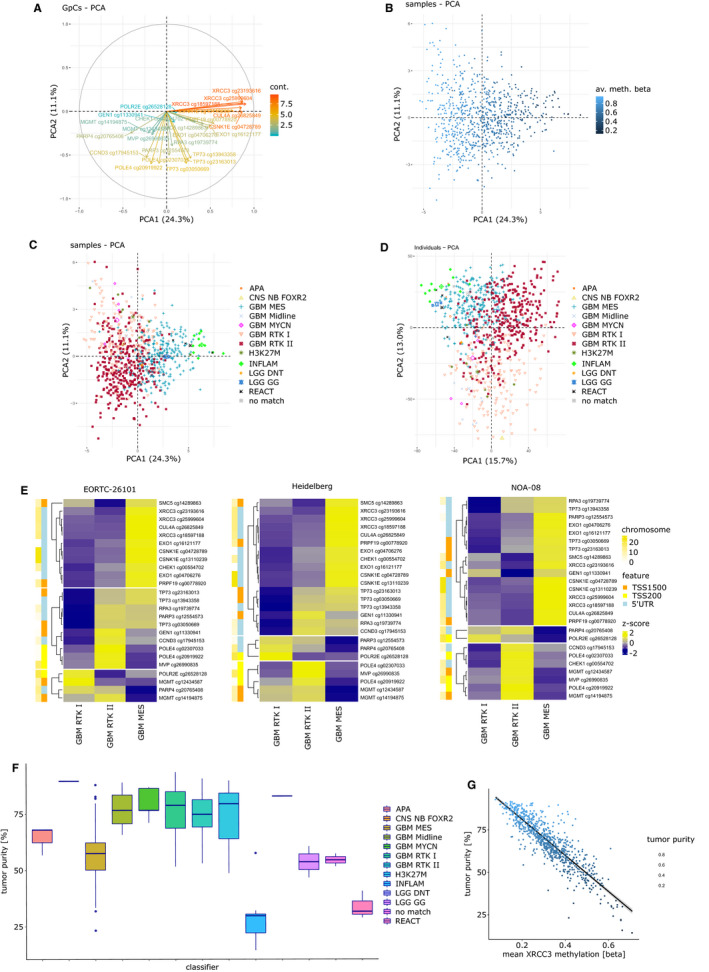
DNA damage response (DDR) methylation profiles. A, Principle component analysis (PCA) showing 5'‐cytosine‐phosphat‐guanine‐3' (CpG) direction. B, PCA with samples colored by Classifier assignment with the 25 DDR CpGs (C) and the 5000 most variable CpGs (D). E, Methylation of DDR CpGs according to the three most abundant glioblastoma subgroups (MES, RTK I, and RTK II). F, Tumor purity according to tumor subtype. G, Example of correlation between tumor purity and XRCC3 methylation. 5ʹ‐UTR, 5ʹ untranslated region; av., average; cont., contribution to a principle component; meth., methylation; TSS200, 0‐200 base pairs upstream of transcription start site, TSS1500: 200‐1500 base pairs upstream of transcription start site; PCA1, principle component 1; PCA2, principle component 2; a full list of classifier abbreviations can be found in the Supporting Information

Principle component analysis of DDR methylation reveals two main directions. Dimension 1 (24.3% variance) is dominated by *XRCC3*, *CUL4A*, and *CSK1E* and dimension 2 (11.1% variance) by *POLE4 and TP73* (Figure 2A). Tumor purity was estimated from methylation data and is strongly associated with dimension 1, highlighting the importance of tumor purity on methylation profiles (Figure 2B). Tumors with mesenchymal (MES) classifier assignment differed from the RTK I and RTK II tumors (Figure 2C,D). We analyzed the association of the methylation of single DDR CpGs with classifier assignments in the study cohorts, with a cutoff *P* < .001 (Figure 2E). Distribution of gene methylation was similar in all three investigated glioblastoma cohorts. Methylation of *POLE4* and *MVP* was highest in RKT II tumors, whereas most other genes including XRCC3 and CSNK1E were hypermethylated in mesenchymal classified tumors. Mean *MGMT* methylation was lowest in mesenchymal tumors; however, highest percentage of *MGMT* unmethylated tumors was found in the RTK I subgroup. Comparison between average DDR methylation and global methylation revealed similar patterns with RTK I tumors having lowest methylation levels (*P* < 2.2 × 10^−16^ for both MES and RTK II against RTK I, Figure S4A,B).

Tumor purity might influence the prognostic effect of CpG methylation. Mesenchymal tumors had an average lower tumor purity compared to RTK I or RTK II tumors (median: 0.57 vs 0.79 and 0.75, *P* < 1 × 10^−57^ for both comparisons, Figure 2F). This may additionally explain the on average lower *MGMT* promoter methylation level. Conclusively, *XRCC3*, *CUL4A*, and *CSK1E* methylation highly correlated with tumor purity (Figure 2G; Figure 4SC‐F).

### 3.2 DDR methylome and interaction with therapy outcome in glioblastoma

A univariate cox proportional hazard model was applied to identify survival associated functional CpGs. There were five genes identified in the exploratory Heidelberg cohort, which were associated with overall survival (OS). Of these, only *MGMT* promoter methylation was correlated with better prognosis consistently in all three studies (Data S1).

In a pooled multivariate cox analysis of all three studies seven genes prove to be independent markers in both OS and progression‐free survival (PFS) after correcting for *MGMT* promoter methylation status and multiple testing (Data S1). Beforehand analysis revealed that the criterion of proportionality was not violated. Further analysis separated by *MGMT* promoter methylation revealed that four of the DDR genes (*TP73*, *CSNK1E*, *EXO1*, and *PRPF19*) showed significant association with OS and PFS only in *MGMT* unmethylated tumors (Data S2). Moreover, after addition of tumor purity into the multivariate model, only *PRPF19* (*P* = .005‐.04, c‐index = 0.71‐0.73) and *TP73* (*P* = .02‐.04, c‐index = 0.71‐0.72) methylation were significantly associated with better OS and PFS (Data S2). C‐indices for multivariate analyses ranged for significant CpGs between 0.66 ± 0.02 and 0.73 ± 0.02 confirming on average good performance of the models (see respective [Link], [Link], [Link], [Link] Data).

The recent NOA‐08 long term study analysis showed worse outcome of patients in the RTK I subgroup in our Heidelberg and the NOA‐08 biomarker cohort^7^ as well as the best prognosis for patients with *MGMT* promoter methylated tumors with a RTK II classifier assignment. Though with a global *P*‐value of .058 not significant, there was a trend also in the EORTC 26101 recurrent glioblastoma study toward worse survival of patients with the low methylated RTK I tumors (Figure 5A). Furthermore, recent preliminary data link EGFRvIII to a better prognosis in *MGMT* methylated tumors.^23^ In concordance, we found EGFRvIII and *EGFR* amplification both highest in the RTK II subgroup (Figure 5C,D) as one potential factor driving chemotherapy sensitivity in RTK II tumors.

Focusing on subtype‐specific CpG markers, we identified only *MGMT* promoter methylation specifically correlating with improved survival in RTK II tumors, *MVP* was prognostic in *RTK* I tumors and no CpG was prognostic in MES subgroup tumors (Data S3).

The NOA‐08 study with the chemotherapy vs radiotherapy regimen additionally offers the chance of finding CpGs that are specifically associated with benefit from either treatment. *MGMT*, *GEN1*, *PARP4,* and *CSNK1E* were found to be associated with better survival in the chemotherapy group, whereas TP73 and *CCND3* methylation were linked to radiotherapy sensitivity in the OS analysis, however, none of these reached significance after correction for multiple testing (Data S4). For PFS, only *MGMT* was prognostic in the chemotherapy group.

### 3.3 The association of TERT promoter mutation with methylation profiles and survival

**FIGURE 3:**
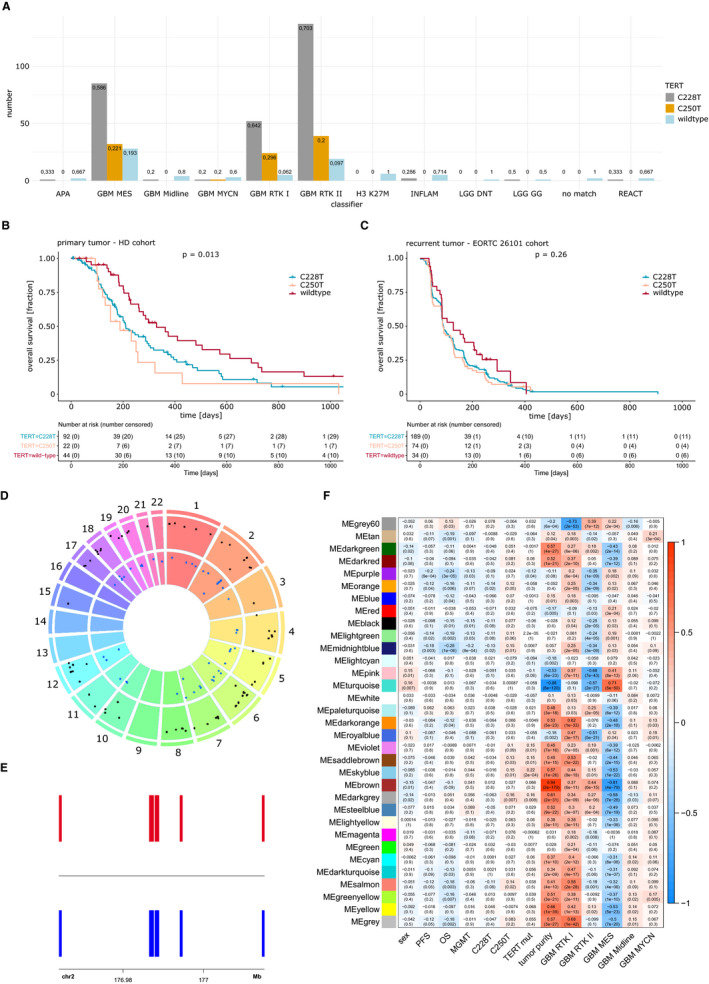
Correlation of telomerase reverse transcriptase (TERT) status with glioblastoma subgroups. A, *TERT* promoter mutation status and tumor classifier subgroup. Survival analysis of patients with primary glioblastoma (B) Heidelberg cohort and recurrent glioblastoma (C) EORTC 26101 cohort. D, Visualization of genome distribution of differential methylated regions between *TERT* promoter mutated and wild‐type tumors. E, Differential methylated regions on chromosome 2 in C225T (upper row) and C250T (lower row) tumors. F, Heatmap of different 5'‐cytosine‐phosphat‐guanine‐3' modules as the result of the WGCNA analysis and relationship to several tumor‐specific markers. C225T, C250T, mutation location upstream of the TERT transcription start site; chr2, chromosome 2; PFS, progression‐free survival; Mb, megabase; OS, overall survival, a full list of classifier abbreviations can be found in the Supporting Information

In the two cohorts with available *TERT* status, *TERT* promoter mutation was found in 377 of 455 (83%) patients. Differences were noted between both cohorts (EORTC 26101 88%, Heidelberg cohort 72%). *TERT* promoter mutation was predominantly found in the three main glioblastoma groups (MES, RTK I, and RTK II). For *TERT* wild‐type tumors 21% belong to these groups, as well as 14% of the *TERT* mutated do (Figure 3A). *TERT* wild‐type status was associated with better PFS in the Heidelberg cohort, but not in the recurrent EORTC 26101 cohort (Figure 3B,C). We restricted the analysis to RTK I, RTK II, MES, Midline, and MYCN^18^ tumors and found 500 differentially methylated CpGs compared to >10 000 differential CpGs in the unrestricted analysis underling the effect of the glioma classification over *TERT* mutation. Differentially methylated regions showed high overlap between C228T and C250T tumors (Figure 3D). On chromosome 2, a cluster of DMRs in the promoter regions of HOXD genes was identified (Figure 3E). A weighted‐gene correlation network analysis (WGCNA) identified 30 modules of CpGs within the EORTC 26101 data set (Figure 3F). Most modules correlated strongly with tumor purity and differentiated between the RTK I, RTK II, and MES subtypes. Highest correlation to *TERT* promoter mutation status was conclusively found in a module specific for the RTK I phenotype with CpGs restricted to chromosome 2 in the *HOXD12*, *HOXD4*, *HOXD3,* and *MIR10B* promoters. However, no specific survival associated module was correlated with *TERT* promoter mutation. Copy‐number analysis revealed association of *TERT* mutation with amplification of chromosome 7 and loss of chromosome 10, for example, with a typical glioblastoma phenotype in differential and WGCNA analysis.

### 3.4 DDR genes and therapy response in glioblastoma cell lines and primary glioma cell cultures

**FIGURE 4:**
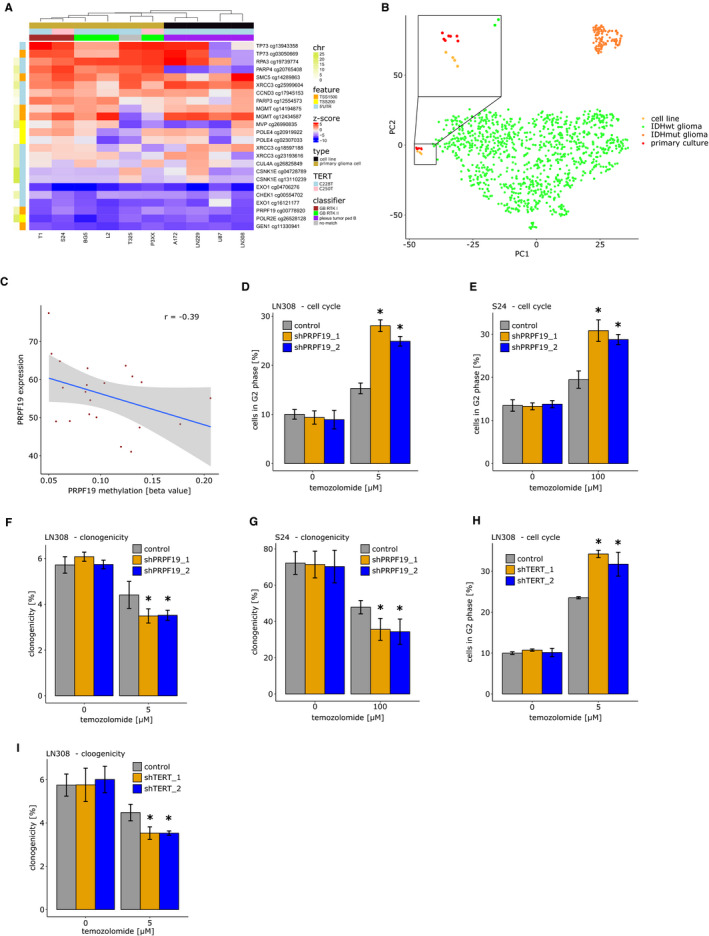
DNA damage response (DDR) genes and therapy response in glioblastoma cells. A, DDR methylome, telomerase reverse transcriptase (TERT) status, *MGMT* promoter methylation and methylation patterns in primary glioblastoma cell cultures and cell lines. B, T‐SNE analysis of cell lines, primary cell cultures and *IDH* wild‐type samples. C, Correlation between *PRPF19* methylation and expression. D, Cell cycle analysis in LN308 cells, silenced for PRPF19 or transfected with respective control vector, after treatment with 5 µmol/L of temozolomide or dimethyl sulfoxide (DMSO) as control. Two different knockdown constructs were used for analysis. E, Cell cycle analysis in S24 cells, silenced for PRPF19 or transfected with respective control vector, after treatment with 100 µmol/L of temozolomide or DMSO as control. Two different knockdown constructs were used for analysis. F, Clonogenicity of LN‐308 cells, silenced for PRPF19 or transfected with respective control vector, after treatment with 5 µmol/L of temozolomide or DMSO as control. Two different knockdown constructs were used for analysis. G, Clonogenicity of S24 cells, silenced for PRPF19 or transfected with respective control vector, after treatment with 100 µmol/L of temozolomide or DMSO as control. Two different knockdown constructs were used for analysis. H, Cell cycle analysis in LN308 cells silenced for TERT or transfected with respective control vector, after treatment with 5 µmol/L of temozolomide or DMSO as control. Two different knockdown constructs were used for analysis. I, Clonogenicity in LN‐308 silenced for TERT or transfected with respective control vector, after treatment with 5 µmol/L of temozolomide or DMSO as control. Two different knockdown constructs were used for analysis. For panels D‐I the mean value and SD of three independent experiments is shown. *P < .05. 5ʹ‐UTR: 5ʹ untranslated region; chr, chromosome; TSS200, 0‐200 base pairs upstream of transcription start site; TSS1500, 200‐1500 base pairs upstream of transcription start site

*TERT* status, DDR methylome, *MGMT* promoter methylation and methylation patterns in primary glioblastoma cell cultures and cell lines are depicted in Figure 4A. Hierarchical clustering separated adherently growing cell lines from primary cells based on DDR methylation. All samples harbor either *TERT* promoter mutation C228T or C250T, all but one (9/10) are *MGMT* methylated (Table S2). Methylation is retained in cell culture as all but one (5/6) primary cell lines show a matching profile with one of the main glioblastoma methylation subgroups. Of note, adherent cell lines grown in serum change their methylation profile in cell culture being most conclusive with a pediatric plexus tumor while copy number variation (CNV) profiles still allow identification as derived from glioblastoma (Table S2; Figure S6). T‐SNE analysis of methylation patterns shows clear separation between cell lines and primary cultures as well as *IDH* wild‐type glioma samples (Figure 4B).

**FIGURE 5:**
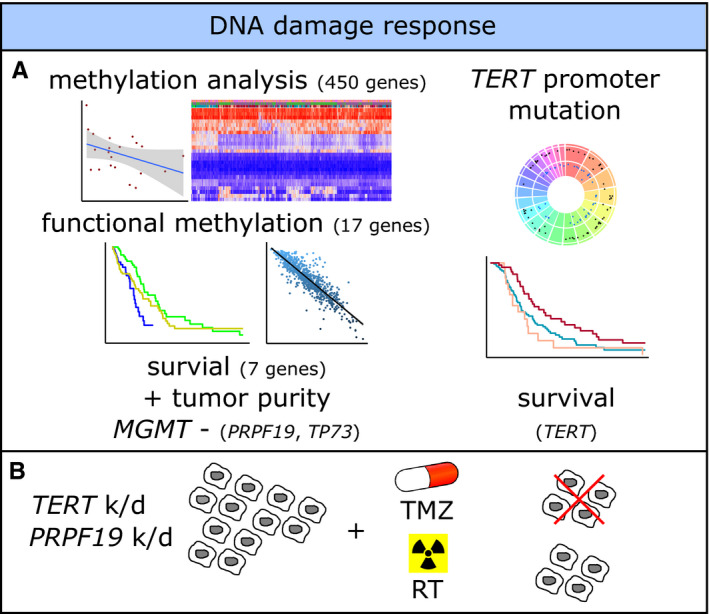
Summary of relevant findings. A, Methylation analysis of 450 DNA damage response (DDR) genes revealed 17 functional DDR genes of which in seven genes hypomethylation showed association with reduced survival. Hypomethylation of *PRPF19* and *TP73* was associated with worse survival in patients with *MGMT* promoter unmethylated tumors with adjustment for tumor purity. *TERT* promoter mutations were correlated with methylation groups and survival times. B, *PRPF19* and *TERT* k/d‐induced sensitivity in glioblastoma cells toward temozolomide but not radiotherapy. k/d, knock down; *MGMT*, unmethylated *MGMT* promoter; TMZ, temozolomide; RT, radiotherapy

*PRFP19* methylation was associated with survival in the combined analysis and in particular in *MGMT* promoter unmethylated, therefore, mainly temozolomide resistant, tumors and retained significance after controlling for tumor purity. Therefore, besides *TERT* promoter mutation, *PRPF19* methylation might be an interesting prognostic marker. We independently confirmed a negative correlation between *PRPF19* methylation and expression (Figure 4C; *r* = −.39) and positive correlation of *TERT* mutation and *TERT* expression in the subset of the Heidelberg cohort with available expression data (Figure S2). Low methylation/high expression primary glioma cultures were picked for lentiviral gene expression modulation of *PRPF19* and *TERT*. Knockdown of *PRPF19* and *TERT* was validated via quantitative real‐time PCR (Figure S7) and resulted in an enhanced response to temozolomide treatment. Cell cycle analysis revealed a higher proportion of G2 arrested cells after temozolomide treatment in tumor cells deficient of *PRFP19* and *TERT* compared to equally treated tumor cells transfected with respective control vector (Figure 4D,E,H). Similarly, clonogenicity of tumor cells treated with temozolomide was reduced particularly in *PRPF19* and *TERT* knockdown cells (Figure 4F,G,I). These effects were not observed for irradiation with both knockdowns (Figure S8). A summary with relevant findings is given in Figure 5A,B.

## 4 DISCUSSION

With this cross‐study analysis based on large recent cohorts of glioblastoma patients, we provide evidence for a prognostic role of DDR genes including DDR methylome and *TERT* promoter mutation status. In our view, this holds several important implications:

Besides the well described DDR gene *MGMT*, we identified DDR genes for which methylation is linked to survival, in particular in patients with *MGMT* promoter unmethylated tumors. This is of particular interest as these tumors at best have very limited response to standard chemotherapy with temozolomide and since further prognostic molecular factors have remained elusive. In our analysis, we identified methylation of the two DDR genes PRPF19 and TP73 significantly associated with better OS and PFS in particular in patients with *MGMT* promoter unmethylated tumors in our multivariate analysis. PRPF19 was previously reported to be involved in DDR^24^ and has a potential role in oncogenesis,^25^ but little is known about its function in glioblastoma. TP73 is a member of the TP53 gene family and overexpressed in a variety of cancers.^26^, ^27^ Its regulation is complex and not fully understood, especially the association between methylation and gene expression is a controversy in different cancers,^26^, ^28^ but a regulatory role in chemotherapy response and probably sensitivity through DNA methylation has been described.^29^ We here describe a negative association between methylation and expression for the prognosis relevant CpG cg13943358 and cg2316013 in glioblastoma. The association in chemotherapy response makes both genes very plausible markers for glioma prognosis in the absence of *MGMT* methylation as a sensitivity factor against chemotherapy, however, formal testing for a predictive effect of the methylation levels of both genes on chemotherapy response was not in the intention of the study and the analysis comparing different treatment methods in the NOA‐08 cohort lacks sufficient sample size for difference detection, and therefore, regarded as exploratory.

Functional evidence for chemosensitivity, however, was validated for PRPF19 by gene silencing in glioblastoma cells. Depletion of *PRPF19* expression resulted in the anticipated sensitization to temozolomide in glioblastoma cell lines and primary cell cultures, and may therefore, be investigated further as a predictive marker in *MGMT* promoter unmethylated glioblastoma. A limitation to use *PRPF19* methylation as prognostic marker is its overall relatively low methylation, but combination with expression may improve prognostic relevance. The TP73 gene was not functionally analyzed in this study, but represents an attractive area for further research. The effect of upfront alkylating agents vs targeted treatments in *MGMT* promoter unmethylated glioblastoma on its prognostic impact could be answered by subgroup analysis of our currently recruiting N^2^M^2^ clinical study.^30^

RTK I glioblastomas remain a less understood, poorly performing group that have lower DDR and overall methylation levels. Only *MVP* methylation was prognostic in this group, however, overall promoter methylation of this gene was low challenging its suitability as a robust marker. Further studies might dive deeper into the differences especially between RTK I and II tumors and their differential methylation profiles.

Further limitations of this methylation analysis approach include the heterogeneity of the three well‐documented patient cohorts used that might reduce sensitivity for markers potentially present only in certain subgroups, but enables to cover a variety of conditions for detection of strong universal markers. Even the NOA‐08 study compared radiotherapy vs chemotherapy the methylation analysis was not powered nor intended to detect chemosensitivity of certain CpGs. Furthermore, the cohorts based on the two large studies EORTC 26101 and NOA‐08 were subsets of the original study population based on the availability of tissue for methylation array analysis. Therefore a sampling bias that often tends toward a better prognosis in the biomarker cohorts cannot be fully excluded. The approach to include only CpGs with negative correlation of methylation with expression in glioblastoma ensures a higher chance of finding functionally relevant genes from a small defined set, but may not be exhaustive as also a subset of CpGs with positive correlations of methylation and expression or CpGs not captured by the stringent threshold could be robust prognostic factors though complex regulations.

*TERT* promotor mutation is the main factor facilitating TERT expression and several studies reported differential outcomes based on *TERT* mutation and *MGMT* promoter methylation,^6^, ^11^, ^12^ this should be taken with caution as these might have included patients with nonglioblastoma methylation groups as a potential confounder. Here, we demonstrated that TERT mutation is associated with worse survival in well characterized cohorts and silencing of *TERT* expression in glioma tumor cells was associated with an enhanced response to temozolomide treatment.

This study furthermore holds implications for preclinical models. Primary glioma cultures nicely retain the glioblastoma‐like methylation state, whereas cell lines change to a methylation profile most consistent with a pediatric plexus tumor. Although we have observed similar results for our functional studies between primary glioma cells and adherent cell lines and both models cluster outside the patient tumor samples, the methylation profiling strongly encourages the use of primary cell lines as an appropriate model glioma for glioma biology as they retain a well‐preserved glioma methylation phenotype. Of note, we have not observed a glioblastoma MES primary cell line in our sample. This might be because of the relatively low number of primary cell lines (n = 9), but the lower tumor purity in MES glioblastomas might prevent detection of this phenotype in cell culture.

In summary, low methylation of DDR genes and *TERT* promoter mutation are associated with worse prognosis in glioblastoma patients and current studies on DDR inhibitors with and without other cytotoxic or immunological therapies may finally yield benefit especially for the heavily underserved patient population with tumors having an unmethylated *MGMT* promoter.

## CONFLICT OF INTEREST

TK, AB, AS, FS, TG, CM, DH, AW, PH, PR, MB, CO, RS, FW, AB, AD, and MP reported no conflict of interest. Michael Weller: Research grants from Abbvie, Adastra, Bristol Meyer Squibb (BMS), Dracen, Merck, Sharp & Dohme (MSD), Merck (EMD), Novocure, Piqur and Roche, and honoraria for lectures or advisory board participation or consulting from Abbvie, Basilea, Bristol Meyer Squibb (BMS), Celgene, Merck, Sharp & Dohme (MSD), Merck (EMD), Novocure, Orbus, Roche and Tocagen. Martin van den Bent: Consulting for Celgene, BMS, Agios, Boehringer, Abbvie, Bayer, Carthera, Nerviano, Genenta. Wolfgang Wick has received study drug support from Apogenix, Pfizer, and Roche and consulted for MSD and Roche with all financial reimbursement to the University Clinic.

## AUTHOR CONTRIBUTIONS

Study concept and design: TK, WW; acquisition of molecular and in vitro data: TK, AB, FS, DH, PR, WW; data acquisition of clinical studies: TK, FS, TG, CM, AW, MW, MB, RS, FW, AB, AD, MP, WW; analysis and interpretation of data: TK, AB, AS, WW; statistical analysis: TK; study supervision and coordination: WW; writing the manuscript: TK, AB, WW; critical revision of manuscript for intellectual content: AS, FS, TG, C.M, DH, AW, PK, PR, MB, CO, MW, MB, RS, FW, AB, AD, MP.

## Supporting information

Data S1

Data S2

Data S3

Data S4

Supplementary Material
