# Supplementary material for: Methylome analyses of three glioblastoma cohorts reveal chemotherapy sensitivity markers within DDR genes
Source: Cancer Med. 2020 Sep 29;9(22):8373–85. doi: 10.1002/cam4.3447 (PMC7666733; doi:10.1002/cam4.3447)
Supplement: Supplementary file 5 — Supplementary Material [file CAM4-9-8373-s005.pdf]

Methylome analyses of three glioblastoma cohorts reveal chemotherapy sensitivity markers within DDR genes

Supporting information including supplementary figures, tables and methods

Tobias Kessler[1,2], Anne Berberich[1,2], Ahmed Sadik[3], Felix Sahm[4,5], Thierry Gorlia[6], Christoph Meisner[7], Dirk Hoffmann[1,2,8], Antje Wick[2], Philipp Kickingereder[9], Petra Rübmann[1], Martin Bendszus[9], Christiane Opitz[3], Michael Weller[10], Martin van den Bent[11], Roger Stupp[12], Frank Winkler[1,2], Alba Brandes[13], Andreas von Deimling[4,5], Michael Platten[14,15], Wolfgang Wick[1,2]

[1]Clinical Cooperation Unit Neurooncology, German Cancer Consortium (DKTK), German Cancer Research Center (DKFZ)

[2]Department of Neurology and Neurooncology Program of the National Center for Tumor Diseases, Heidelberg University Hospital

[3]Brain Tumor Metabolism, DKTK, DKFZ

[4]Department of Neuropathology, Heidelberg University Hospital

[5]Clinical Cooperation Unit Neuropathology, DKTK, DKFZ

[6]European Organization for Research and Treatment of Cancer Headquarters, Brussels, Belgium

[7]Institute für Clinical Epidemiology and Applied Biometry, Tübingen, Germany

[8]Faculty of Biosciences, Heidelberg University, Heidelberg, Germany

[9]Department of Neuroradiology, Heidelberg University Hospital

[10] Department of Neurology, University Hospital and University of Zurich, Zurich, Switzerland

[11] the Brain Tumor Center at, Erasmus MC Cancer Institute, Rotterdam, Netherlands

[12]Northwestern University, Feinberg School of Medicine, Chicago, IL, USA

[13]Department of Medical Oncology, Azienda USL-IRCCS Institute of Neurological Sciences, Bologna, Italy

[14]Clinical Cooperation Unit Neuroimmunology and Brain Tumor Immunology, DKTK, DKFZ, all Heidelberg, Germany

[15]Department of Neurology, Medical Faculty Mannheim, Heidelberg University, Mannheim, all Germany

Supplementary Figures

Suppl. Figure S1

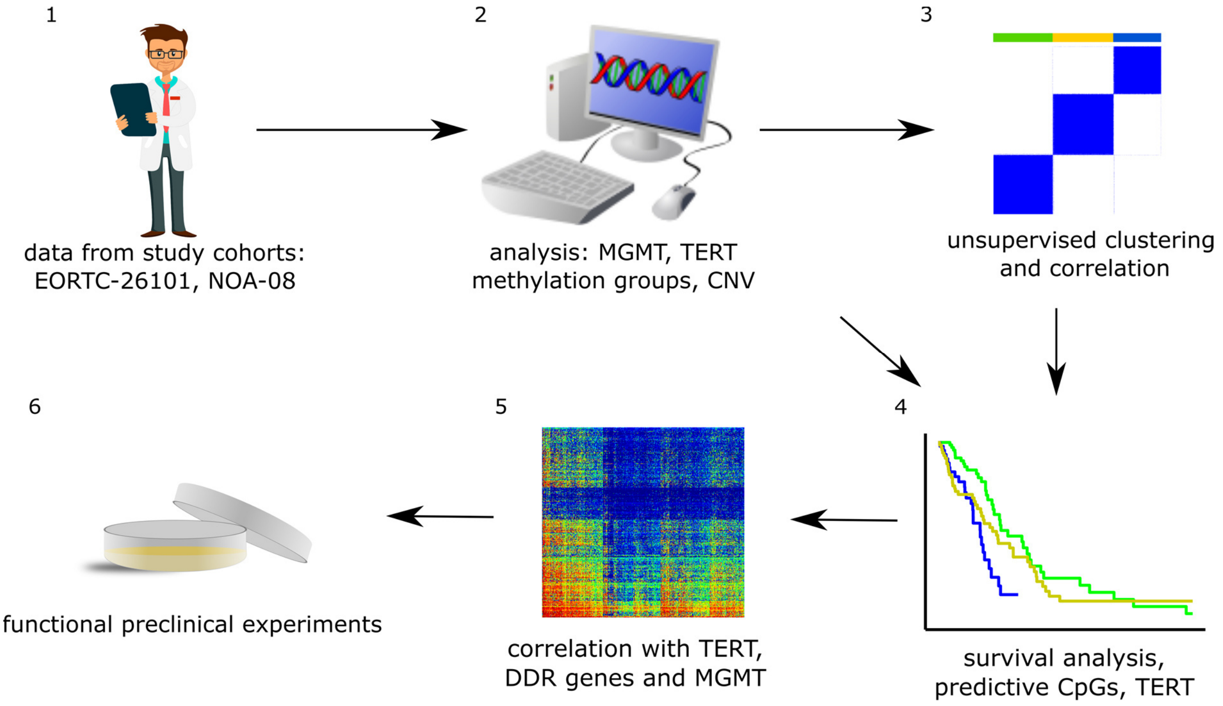

Suppl. Figure S1: Workflow of the project.

Suppl. Figure S2

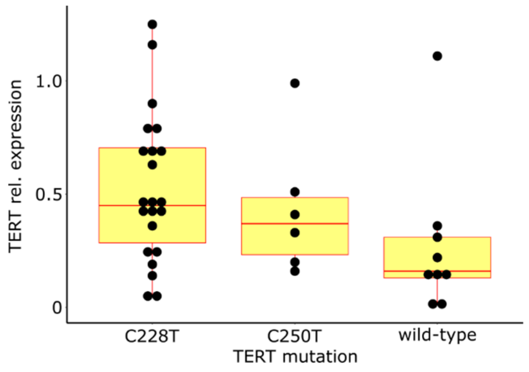

Suppl. Figure S2: *TERT* expression according to *TERT* mutation status. Figure shows TERT expression measured by RNA sequencing in the subset of the Heidelberg cohort with available RNA expression and DNA sequencing data (n = 37).

C225T, C250T: mutation location upstream of the TERT transcription start site, rel: relative

Suppl. Figure S3

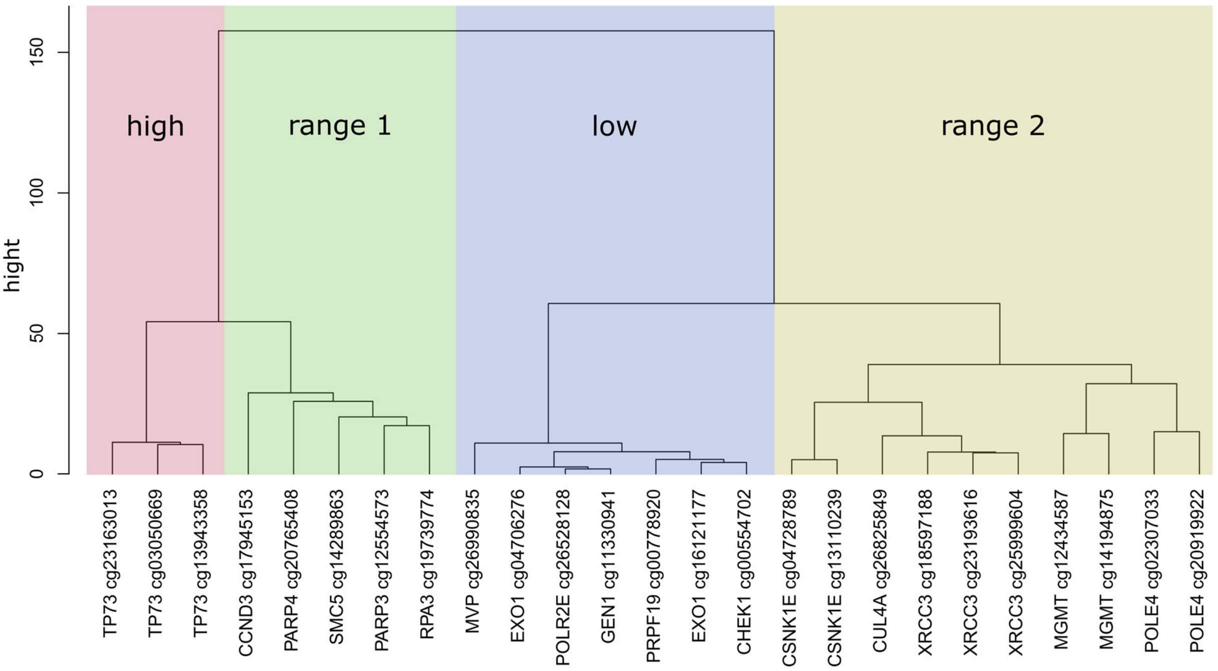

Suppl. Figure S3: Hierarchical clustering of DDR CpG methylation. Hierarchical clustering of DDR CpG methylation shown for the all samples of the three study cohorts combined (NOA-08: *n* = 104, EORTC26101: *n* = 297, Heidelberg cohort: *n* = 298). The "cg" numbers behind each gene correspond to the CpGs in the methylation array.

Suppl. Figure S4

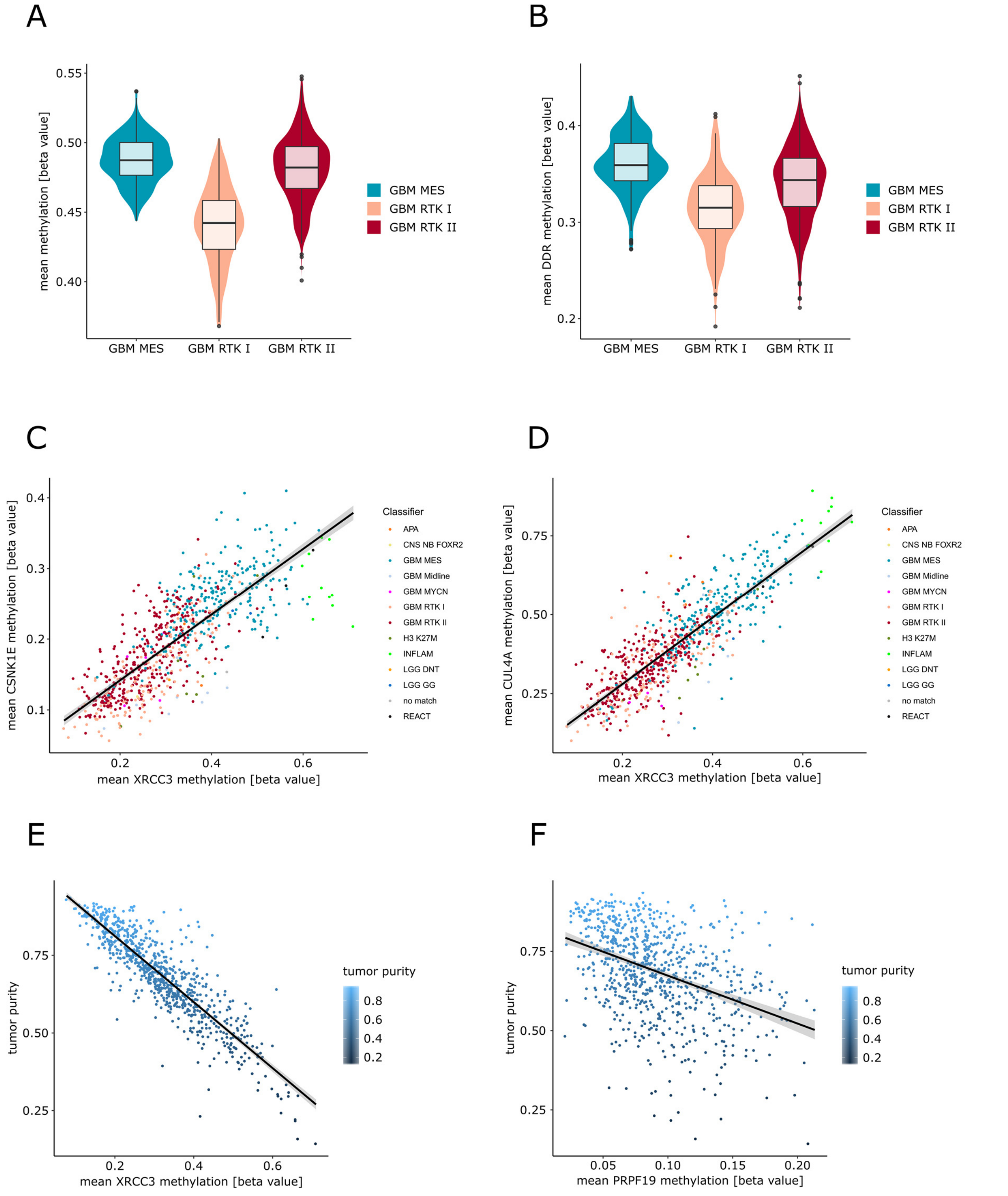

Suppl. Figure S4: Mean methylation in glioma subgroups and CpG correlation with tumor purity. **(A)** Mean CpG methylation according to methylation classifier assignment. **(B)** Mean methylation of functional DDR CpGs according to classifier assignment. **(C-F)** Correlation blots between selected functional DDR CpG methylation and tumor purity.

*a full list of classifier abbreviations can be found in the supporting information.

Suppl. Figure S5

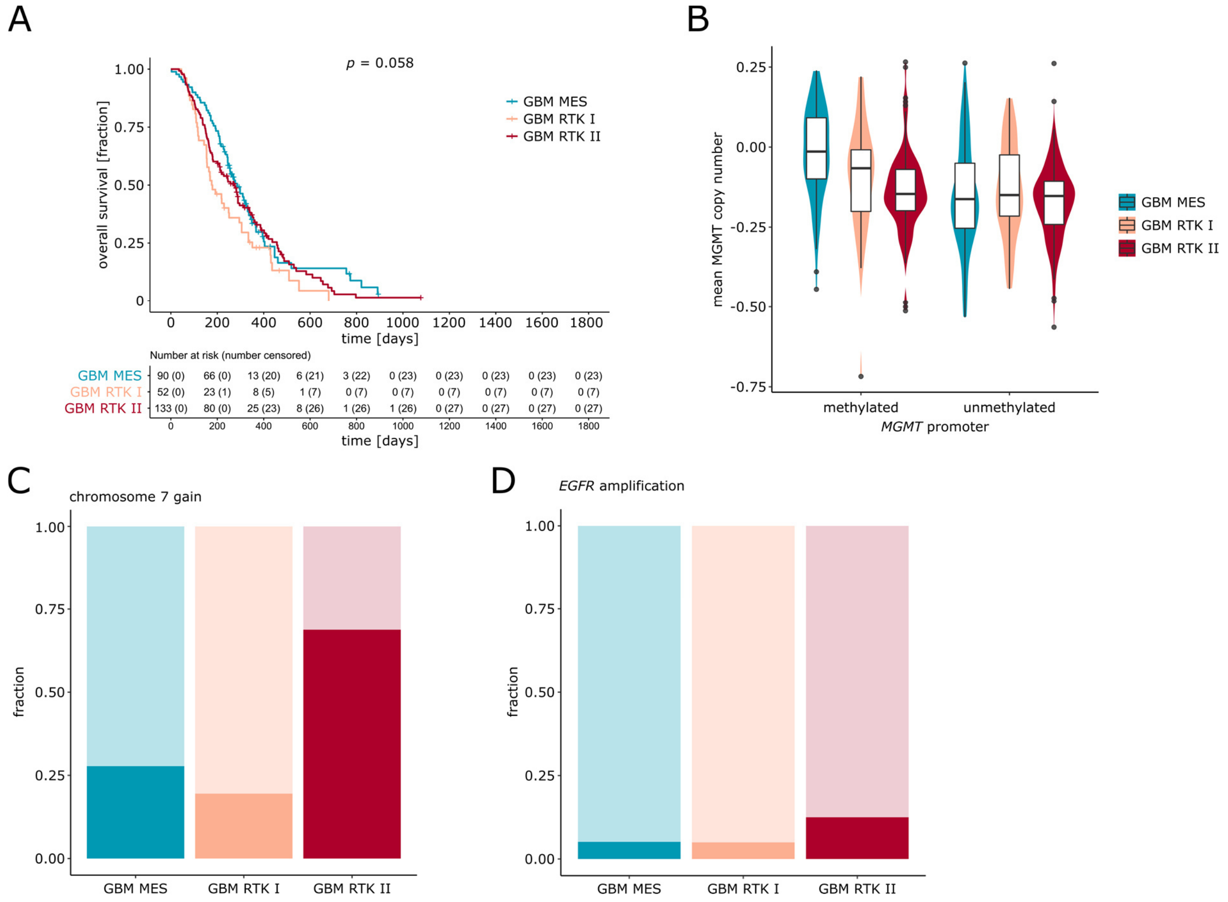

Suppl. Figure S5: Association of glioma subgroups with survival and chromosome 7 alterations. (A) Survival curves of patients from the EORTC-26101 study according to methylation classifier assignment. **(B)** *MGMT* copy number variation according to *MGMT* promoter methylation and methylation classifier assignment. **(C)** Chromosome 7 gain and **(D)** *EGFR* amplification according to methylation classifier assignment.

*a full list of classifier abbreviations can be found in the supporting information.

Suppl. Figure S6

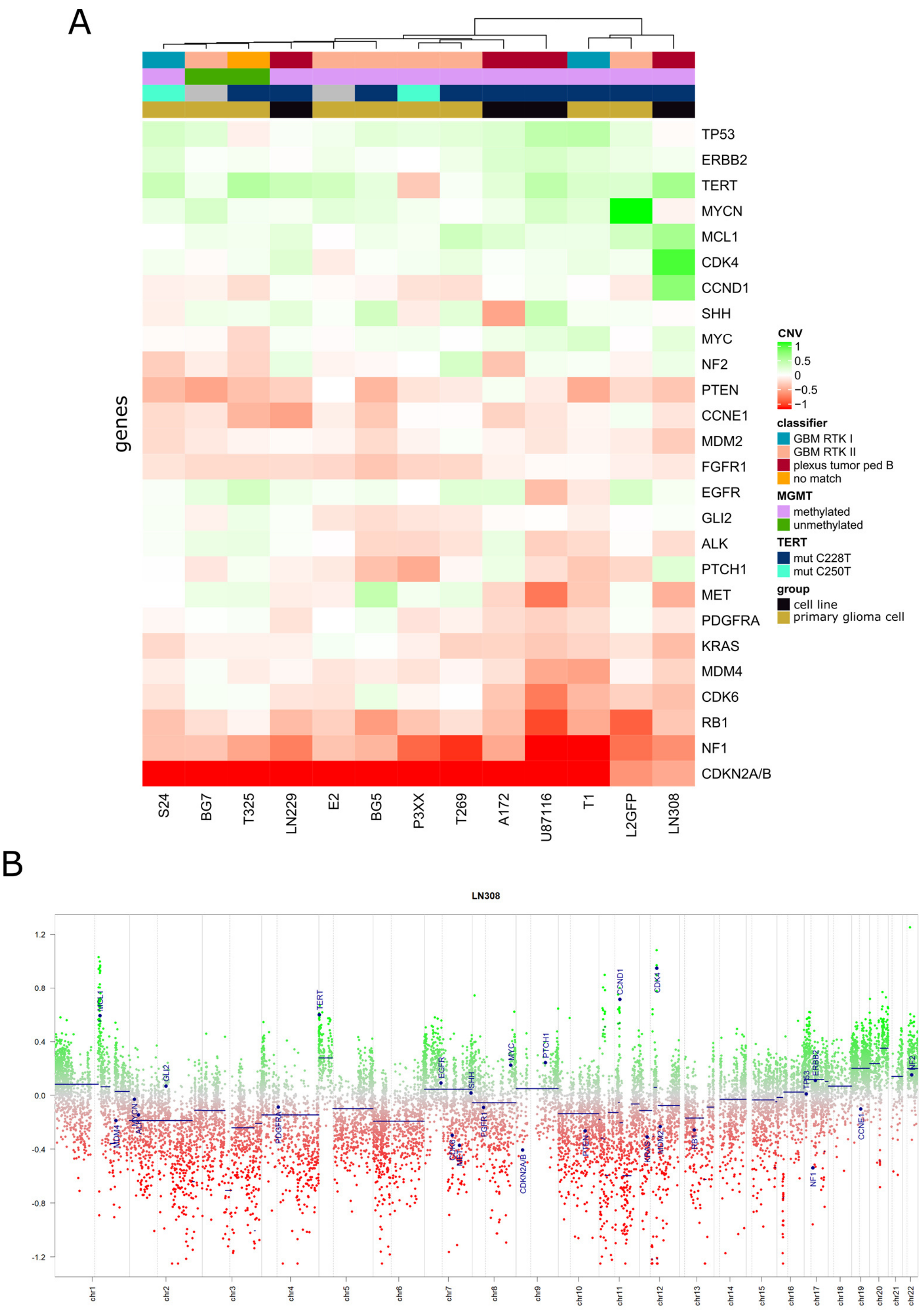

Suppl. Figure S6: Copy number variations of glioma cell lines and primary cultures. (A) Heatmap of copy number alterations of 26 important glioma genes of glioma cell lines (n = 4) and primary cell cultures (n = 9) determined with methylation EPIC array. **(B)** Example of a copy number profile of the glioma cell line LN308.

Suppl. Figure S7

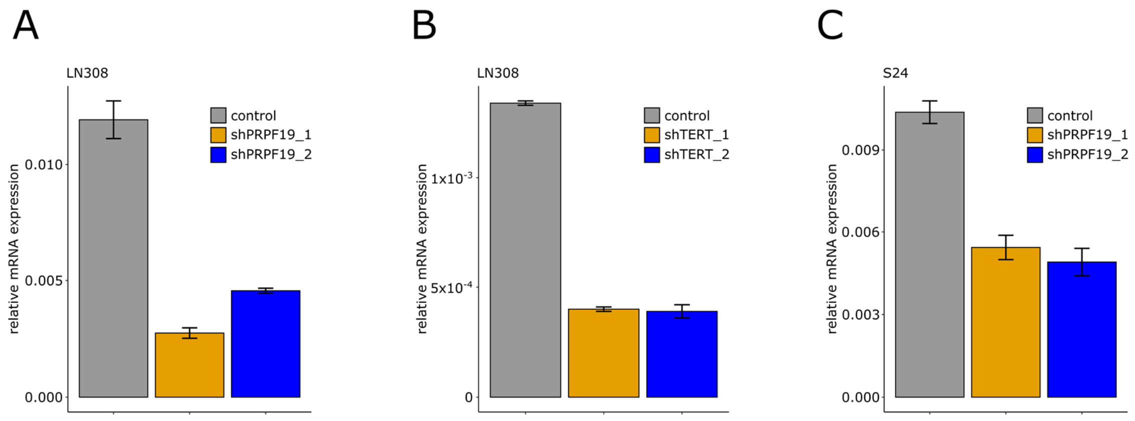

Suppl. Figure S7: Knockdown validation with Quantitative real-time PCR (qRT-PCR). (A-C) RNA expression measured with qRT-PCR of *PRPF19* and *TERT* in glioma cell lines and primary glioma cells transfected with a vector control and two different knockdown constructs per gene (shPRPF19_1, shPRPF19_2, shTERT_1 and shTERT_2).

Suppl. Figure S8

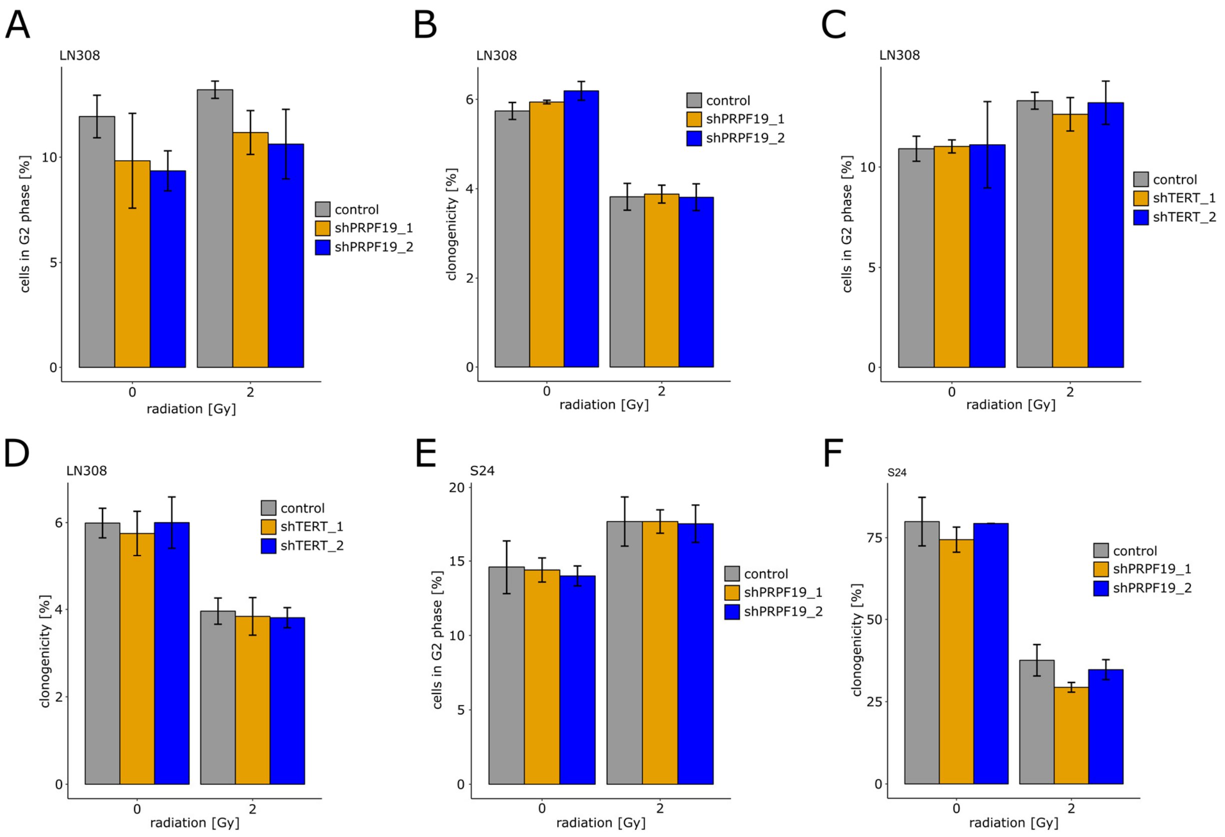

Suppl. Figure S8: Tumor cell response to radiotherapy according to *PRPF19* and *TERT* knockdown. (A, C, E) Percentage of tumors cells in the G2 phase in cell cycle analysis for vector control and two knockdown constructs treated with 2 Gy radiation or control treatment. **(B, D, F)** Clonogenicity of tumor cells transfected with vector control and two knockdown constructs and treated with 2 Gy of radiation or control treatment. All panels in this figure represent the mean value and SD of three independent experiments.

Supplementary Tables

Suppl. Table S1

Suppl. Table S1: List of functional CpGs in high grade *IDH* wildtype glioma

GpG#	Gene	Correlation (r-value)	Adjusted *p*-value
cg17945153	CCND3	-0.532	0.0303
cg00554702	CHEK1	-0.507	0.0428
cg13110239	CSNK1E	-0.533	0.0303
cg04728789	CSNK1E	-0.514	0.0394
cg26825849	CUL4A	-0.497	0.0494
cg16121177	EXO1	-0.514	0.0394
cg04706276	EXO1	-0.500	0.0468
cg11330941	GEN1	-0.504	0.0458
cg12434587	MGMT	-0.663	0.0008
cg14194875	MGMT	-0.633	0.0024
cg26990835	MVP	-0.512	0.0396
cg12554573	PARP3	-0.547	0.0262
cg20765408	PARP4	-0.500	0.0468
cg12290764	POLE4	-0.539	0.0285
cg20919922	POLE4	-0.603	0.0057
cg02058002	POLE4	-0.548	0.0260
cg02307033	POLE4	-0.561	0.0207
cg26528128	POLR2E	-0.542	0.0278
cg00778920	PRPF19	-0.538	0.0285
cg19739774	RPA3	-0.511	0.0396
cg14289863	SMC5	-0.620	0.0034
cg13943358	TP73	-0.536	0.0287
cg23163013	TP73	-0.619	0.0034
cg03050669	TP73	-0.542	0.0278
cg06830875	TP73	-0.500	0.0468
cg18597188	XRCC3	-0.537	0.0285
cg23193616	XRCC3	-0.526	0.0343
cg25999604	XRCC3	-0.503	0.0468

Suppl. Table S2

Suppl. Table S2: Molecular characteristics of glioma cell lines and primary cultures

**Name**	**Type**	**Classifier**	***MGMT***	***TERT* mut**	***CNV***
A172	Cell line	Plexus tumor	M	C228T	Gain of chr 19q, loss of chr 4q, 6, 8, 11q, 14, 18q, X, CDKN2A/B del
BG5	Primary culture	GB RTK I	M	C228T	Gain of chr 7, loss of chr 10, CDKN2A/B del
L2	Primary culture	GB RTK II	M	C228T	Gain of Chr 7, loss of chr 1q, 13q
LN229	Cell line	Plexus tumor	M	C228T	Gain of chr 7, 17q, 20, 21 loss of chr 6q, 11, CDKN2A/B del
LN308	Cell line	Plexus tumor	M	C228T	Gain of chr 5p, 7p, 20q, CDK4 amp
P3XX	Primary culture	GB RTK II	M	C250T	Gain of chr 7, 10p, 19, 20, loss of chr 4, 9, 19q, X, CDKN2A/B del
S24	Primary culture	GB RTK I	M	C250T	Gain of chr 20, loss of chr 6, 8 and 10, CDKN2A/B del
T1	Primary culture	GB RTK I	M	C228T	Gain of chr 7, loss of chr 10, CDKN2A/B del
T325	Primary culture	No match	U	C228T	Gain of chr 7, loss of chr 4q, 5q, 6, 8, 10 and 11, CDKN2A/B del
U87116	Cell line	Plexus tumor	M	C228T	Gain of chr 7q, 13q, 20q, loss of chr 4q, 6p, 9p, 11p, 12p, 13p, 14p, CDKN2A/B del
T269	Primary culture	GB RTK II	M	C228T	Gain of chr 1, 3, 5, 7, 9, 12-15, 18-20, 22, CDKN2A/B del

CNV: copy number variation, M: promoter **m**ethylated, U: promoter **u**nmethylated, C225T, C250T: mutation location upstream of the TERT transcription start site, chr: chromosome, amp: amplification, del: deletion.

Supplementary Methods

Cell culture

The human glioblastoma cell lines U87MG, A172, LN308 (ATCC; Manassas, USA) and LN229 (N. de Tribolet, Lausanne, Switzerland), were kept in complete medium composed of Dulbecco's modified Eagle Medium (DMEM, High glucose, 4.5 g/l, Sigma-Aldrich, St. Louis, USA) supplemented with 10% FBS and 1% penicillin/streptomycin (Sigma-Aldrich). The primary glioblastoma cell cultures (glioma initiating cell cultures, GICs) S24, T1, T269 and T325 were established from freshly dissected glioblastoma tissue from adult patient after informed consent[1]. Primary glioma cell cultures P3XX and BG5 were kindly provided by H. Miletic, K. G. Jebsen Brain Tumour Research Centre, University of Bergen. Primary glioma cell culture L2 was kindly provided by F. Siebzehnrubl, European Cancer Stem Cell Research Institute, Cardiff University. Primary glioma cell culture E2 was kindly provided by C. Watts, John van Geest Centre for Brain Repair, University of Cambridge. Primary glioma cell lines were cultured in neurosphere medium (DMEM/F12 medium (Life Technologies, Carlsbad, USA) enriched with B27 supplement, heparin (5 µg/ml), basic fibroblast growth factor (bFGF) (20 ng/ml) and epidermal growth factor (EGF) (20 ng/mL).

Reagents

Temozolomide (Merck, Darmstadt, Germany, catalogue number: T2577 [Sigma-Aldrich]) was dissolved in DMSO. Cells were treated after seeded with temozolomide or respective DMSO control. As the primary S24 cell cultures were dissected from recurrent glioblastoma, higher doses of temozolomide were necessary to obtain reliable effects of temozolomide treatment. In addition, for cell cycle analysis S24 cells were treated daily for 72 h with temozolomide.

Generation of lentiviral shRNA knockdown cells

Knockdown of *PRPF19* and *TERT* gene expression was performed using commercial shERWOOD ultramiR lentiviral small hairpin RNAs (shRNA) with pZIP-SFFV-turboRFP-Puro as vector. Overall, three different shRNA constructs were tested and the two most effective constructs were used for further experiments (for PRPF19: ULTRA-3272605, ULTRA-3272606, ULTRA-3272601, for TERT: ULTRA-3380692, ULTRA-3380693, ULTRA-3380694, transOMIC technologies inc., Huntsville, USA). A non-targeting RFP tagged shRNA lentiviral construct was used as vector control. Cells were selected after transduction by FACS sorting (FACS Canto II, BD) of RFP positive cells and further kept under selection with puromycin for culturing conditions.

TERT promoter mutation analysis

TERT promoter mutation analysis was performed as described before[2]. DNA was extracted from FFPE material. Areas with highest available tumor content were chosen. Extraction was carried out using the automated Maxwell system (Promega, Madison, WI, USA). For polymerase chain reaction (PCR), 20 ng of DNA and KOD Hot Start Master Mix (Merck, Darmstadt, Germany) were employed. Briefly, PCR was performed in a total reaction volume of 20 µL and was started with an initial polymerase activation step at 95°C for 2 minutes, followed by 35 cycles beginning with denaturation at 95°C for 20 s, annealing for 45 s at 62°C temperatures, and extension at 70°C for 10 s, followed by a final extension at 70°C for 20 minutes with subsequent cooling to room temperature. The amplification product (2 µL) was submitted to bidirectional sequencing using the BigDye Terminator v3.1 Sequencing Kit (Applied Biosystems, Foster City, CA, USA). Mutations were identified by visual analysis of the sequence chromatograms using Sequence Pilot version 3.1 software (JSI-Medisys, Kippenheim, Germany). Primers used for detecting *TERT* promoter mutations are indicated below.

Forward (5′-3′): CAGCGCTGCCTGAAACTC, reverse (5′-3′): GTCCTGCCCCTTCACCTT.

Quantitative real-time PCR (qRT-PCR)

Total RNA was extracted using a RNA purification system (Qiagen, Hilden, Germany) and cDNA was synthesized using High-Capacity cDNA Reverse Transcription kit (Applied Biosystems, Carlsbad, USA). qRT-PCR was performed according to standard protocols in an ABI 7000 thermal cycler using primaQUANT qPCR-CYBR-Green Mastermix (Steinbrenner, Wiesenbach, Germany). Standard curves were generated for each gene and the amplification was 90–100% efficient. Relative quantification of gene expression was determined by comparison of threshold values. All results were normalized to glyceraldehyde-3-phosphate dehydrogenase (GAPDH).

Primer Sequences:

**qRT-PCR**	**forward (5´- 3´)**	**reverse (5´- 3´)**
human *GAPDH*	CTCTCTGCTCCTCCTGTTCGAC	TGAGCGATGTGGCTCGGCT
human *TERT*	TGTGCACCAACATCTACAAG	GCGTTCTTGGCTTTCAGGA
human *PRPF19*	ATGTCCCTAATCTGCTCCATCT	GAGCCGCCGCTCATAAACA

Clonogenicity assay

Clonogenic capacity (“clonogenicity”) of glioblastoma cells was analyzed by limiting dilution assay (LDA). For that, cells were seeded in 96-well microplates with 300, 50, 8.3 and 1.4 cells in 0.2 ml of culture medium per well. Cells were treated after seeding with temozolomide or respective DMSO amounts as indicated in the figures, or irradiated with 2 or 4 Gray radiotherapy. After three weeks, plates were analyzed for wells showing clones and clonal frequency was calculated using extreme limiting dilution (ELDA) software [3].

Cell cycle assay

For cell cycle, analysis 150,000 cells (LN308) or 300.000 cells (S24) were seeded in 6-well plates and treated with the indicated concentrations of temozolomide or DMSO. For assays with radiotherapy, cells were irradiated with 2 or 4 Gray. After 72h cells were separated into single cells, incubated in

70% ethanol for 1h and stained with 40 µg/ml propidium iodide enriched with 20 µg/ml RNAse for 30min. FACS analysis was performed using a BD-FACS Canto II flow cytometer. Final data were processed with FloJo flow cytometry analysis software (Treestar).

Dimensionality reduction, clustering and correlation network analysis

Principle component analysis (PCA) is a common method for linear dimensionality reduction that can visualize high dimensional data in a two-dimensional diagram using principle components[4]. We implanted PCA in R with the function “prcomp”, and visualization was done with the package “ggplot2” (version 3.2.1). Unsupervised clustering was performed using the package “ConsensusClusterPlus” (version 1.48.0) in R.

A weighted protein correlation network analysis (WPCNA) can identify clusters of methylation sites that show high intensity correlation and therefore similar concurrent regulation[5]. Subsequent association with clinical variables identifies correlations between CpG clusters and variables. We implemented the WGCNA with the R package “WGCNA” (version 1.68) with minor adaptations for the use in methylation analysis. The full methylation dataset with 450,000 CpGs per sample was used for WGCNA. Soft threshold was automatically picked based on the criterion of approximate scale-free topology, and the network type was defined as signed hybrid. Block wise consensus module detection was performed on a 200 GB RAM, 28 core cluster using a block size of 40,000. Associations with cluster assignments were calculated using the package “globaltest” (version 5.38.0). All R scripts are customized implementations of above-mentioned packages, and the source code can be made available upon reasonable request.

Supplementary References

1. Lemke D, Weiler M, Blaes J, et al. Primary glioblastoma cultures: can profiling of stem cell markers predict radiotherapy sensitivity? J Neurochem. 2014;131: 251-264.

2. Koelsche C, Hovestadt V, Jones DT, et al. Melanotic tumors of the nervous system are characterized by distinct mutational, chromosomal and epigenomic profiles. Brain Pathol. 2015;25: 202-208.

3. Hu Y, Smyth GK. ELDA: extreme limiting dilution analysis for comparing depleted and enriched populations in stem cell and other assays. J Immunol Methods. 2009;347: 70-78.

4. Jolliffe IT, Cadima J. Principal component analysis: a review and recent developments. Philos Trans A Math Phys Eng Sci. 2016;374: 20150202.

5. Horvath S, Dong J. Geometric interpretation of gene coexpression network analysis. PLoS Comput Biol. 2008;4: e1000117.

Methylation classifier abbreviations

Below the abbreviations of methylation classifier groups in this study are listed. The full current list of current brain tumor methylation classifier groups can be downloaded from: https://www.molecularneuropathology.org/

APA: Methylation class anaplastic pilocytic astrocytoma

CNS NB FOXR2: Methylation class CNS neuroblastoma with FOXR2 activation

GBM MES: Methylation class glioblastoma, IDH wildtype, subclass mesenchymal

GBM Midline: Methylation class glioblastoma, IDH wildtype, subclass midline

GBM MYCN: Methylation class glioblastoma, IDH wildtype, subclass MYCN

GBM RTK I: Methylation class glioblastoma, IDH wildtype, subclass RTK (receptor tyrosine kinase) I

GBM RTK II: Methylation class glioblastoma, IDH wildtype, subclass RTK II

H3K27M: Methylation class diffuse midline glioma H3 K27M mutant

INFLAM: Methylation class control tissue, inflammatory tumor microenvironment

LGG DNT: Methylation class low grade glioma, dysembryoplastic neuroepithelial tumor

LGG GG: Methylation class low grade glioma, ganglioglioma

REACT: Methylation class control tissue, reactive tumor microenvironment

Plexus tumor ped B: Methylation class plexus tumor, subclass pediatric B
